# Quantitative descriptions of rice plant architecture and their application

**DOI:** 10.1371/journal.pone.0177669

**Published:** 2017-05-17

**Authors:** Xumeng Li, Xiaohui Wang, Yulin Peng, Hailin Wei, Xinguang Zhu, Shuoqi Chang, Ming Li, Tao Li, Huang Huang

**Affiliations:** 1 Agricultural mathematical modeling and data processing center, Hunan Agricultural University, Changsha, China; 2 International Rice Research Institute, Metro Manila, Philippines; 3 State Key Laboratory of Hybrid Rice, Changsha, China; 4 Hunan Agricultural University, Changsha, China; The National Orchid Conservation Center of China; The Orchid Conservation & Research Center of Shenzhen, CHINA

## Abstract

Plant architecture is an important agronomic trait, and improving plant architecture has attracted the attention of scientists for decades, particularly studies to create desirable plant architecture for high grain yields through breeding and culture practices. However, many important structural phenotypic traits still lack quantitative description and modeling on structural-functional relativity. This study defined new architecture indices (AIs) derived from the digitalized plant architecture using the virtual blade method. The influences of varieties and crop management on these indices and the influences of these indices on biomass accumulation were analyzed using field experiment data at two crop growth stages: early and late panicle initiation. The results indicated that the vertical architecture indices (LAI, PH, 90%-DRI, MDI, 90%-LI) were significantly influenced by variety, water, nitrogen management and the interaction of water and nitrogen, and compact architecture indices (H-CI, Q-CI, 90%-LI, 50%-LI) were significantly influenced by nitrogen management and the interaction of variety and water. Furthermore, there were certain trends in the influence of variety, water, and nitrogen management on AIs. Biomass accumulation has a positive linear correlation with vertical architecture indices and has a quadratic correlation with compact architecture indices, respectively. Furthermore, the combination of vertical and compact architecture indices is the indicator for evaluating the effects of plant architecture on biomass accumulation.

## Introduction

Rice is a staple food for more than half of the world population. Improving plant architecture for developing high yield rice varieties has attracted much attention of researchers for decades. Plant architecture is a complex trait that varies among agronomic practices and plays an important role to determine plant tolerance to environmental stress, plant adaptability, harvest index and potential grain yield [1–5]. Architecture is cultivar dependent [1–2] and can be modified by environmental factors, such as light, temperature, humidity and nutrient status [6–10].

In recent years, many important genes/quantitative trait loci controlling plant architecture have been isolated and functionally characterized [11]. For example, the gene *MOC1*was defined as a control for rice tiller bud initiation and outgrowth [12]. The gene *TAC* controls tiller angle [13]. The *D3*, *D10*, *D14*, *D17*, *D27* and *D53*genes are involved in the synthesis or signaling pathways of strigolactones and influence rice plant height [14–18]. The gene nrl1reduces leaf width and semi-rolled leaves [19]. The RTFL genes are involved in the mediation of leaf blade length [20]. The overexpression of the OsAGO7 gene results in upward curling of leaves [21]. In addition to the effort of genetic studies, the effects of various agronomic practices (e.g., water and fertilizer application, transplanting density, planting date and mulching mode, etc.) have also been investigated in rice architecture and yield and the interaction between physiology and architecture [22–25]. Plant architecture can be characterized by a set of traits with complex interactions. In contrast with the favorable case where a single phenotypic trait is influenced by multiple genes and multiple environmental factors, thus providing multiple ways of control, the case where a single gene or environmental factor influences multi-phenotypic traits makes it more complex to work on improving these traits. Therefore, concerning plant architecture, it is crucial to have quantitative descriptions of its different components for effectively investigating structural phenotypic traits of plant architecture. However, many structural phenotypic traits still have not been quantitatively described yet. For example, to the best of our knowledge no quantitative indices to describe the compact/loose degree of plant architecture that nevertheless is extensively used in breeding selection and agronomy studies [26–27].

Three-dimensional digital plant architecture and a virtual blade method can be used to comprehensively and synthetically investigate structural phenotypic characteristics by extracting traits from digital plant architecture. This study aimed to define several architectural indices to describe the plant architecture and analyze the effects of cultivar and water and nitrogen application on architectural indices and the effect of architectural indices on biomass accumulation.

## Materials and methods

### Field experiment design

Field experiments were conducted in lowland and upland fields at the research farm of the International Rice Research Institute (IRRI) located in Los Baños, Philippines (21.25E, 14.18N, 21 m elevation) in 2015. Experiments were implemented in a split-plot design with three replicates. Main plots included three nitrogen fertilizer application rates (N1: 0, N2: 170, and N3: 240 kg ha^−1^). The subplots (42 m^2^) were assigned to two varieties (V1: NSICRc124H, and V2: NSICRc222). There were two water management practices implemented, including fully irrigated plants in the lowland (W1) and alternative water and dry (W2) at the upland experimental site. Rice seeds were sown on 19-Dec-2014. The 24 day-old seedlings were transplanted at a space of 20 cm × 20 cm with two seedlings per hill. Phosphorus (calcium superphosphate) was applied at 64 kgha^−1^ as a basal application. Potassium (potassium chloride) was applied at 148 kg ha^−1^with 50% as basal at transplanting and 50% at the panicle initiation (PI) stage (35 days after transplanting). Nitrogen (urea) was applied in four splits with 19% of the total amount applied as basal, 36% at the mid-tillering (14 days after transplanting), 36% at PI and 9% at heading (59 days after transplanting). In W1, surface water depth was maintained at 3–5 cm in the field from transplanting to 10 days before plant maturity. Diseases, weeds and pests were well-controlled for all experiments. Data of dry biomass and plant architecture was measured on 27-February-2015 (early PI) and 10-March-2015 (late PI, see S1 and S2 Data, S2 File), and biomass accumulation was the difference of dry biomass between the late and early PI stages.

### Digital construction and trait extraction of plant architecture

**Fig 1:**
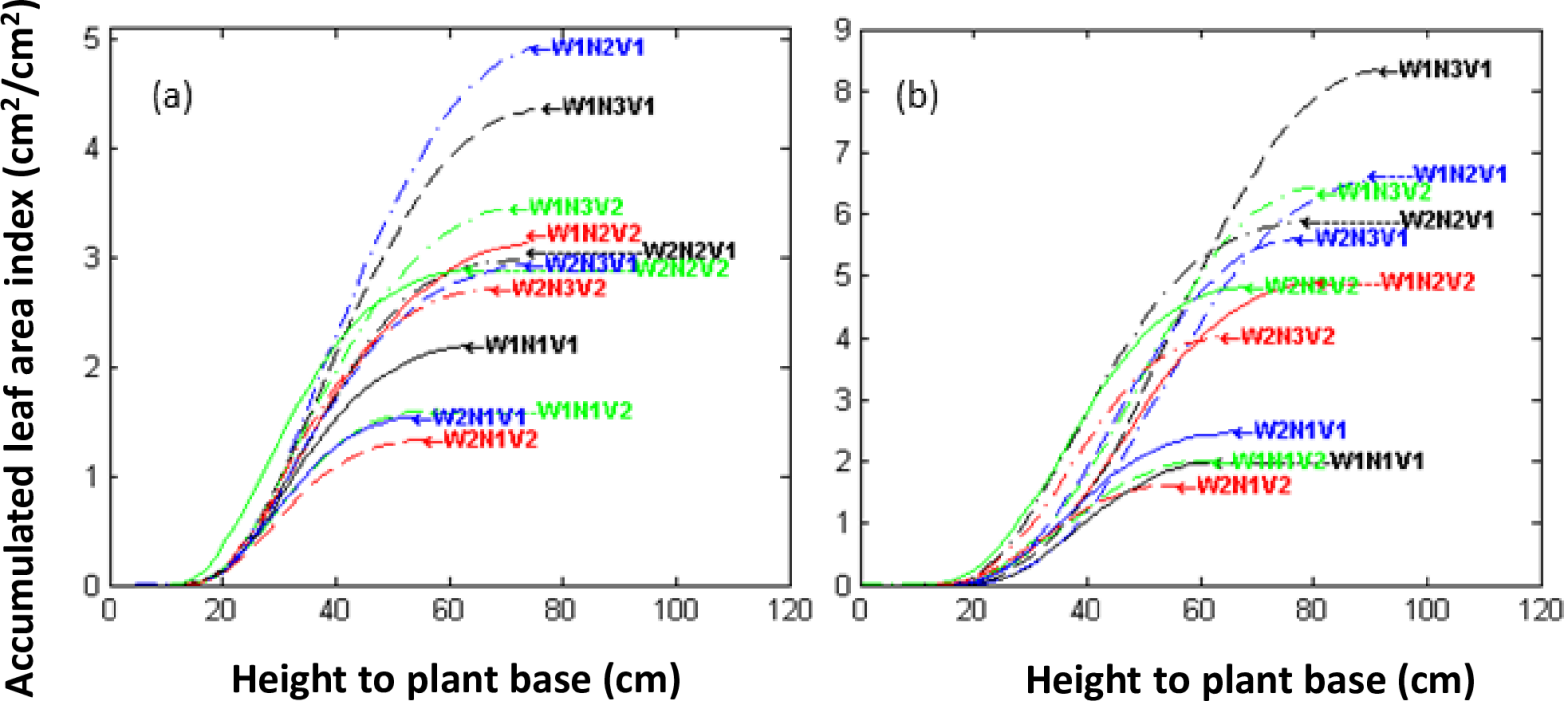
Accumulated leaf area index of leaf layers at the plant height (i.e. along the Z-axis). WiNjVk denoted the treatment which water management practices is Wi, nitrogen fertilizer application rate is Nj, variety is Vk, while the treatment number denoted by i and k varying from 1 to 3, j varying between 1 and 2. VALAI(z) was derived from overlapped digital plant architecture of three representative hills. (a: Early PI; b: Last PI).

**Fig 2:**
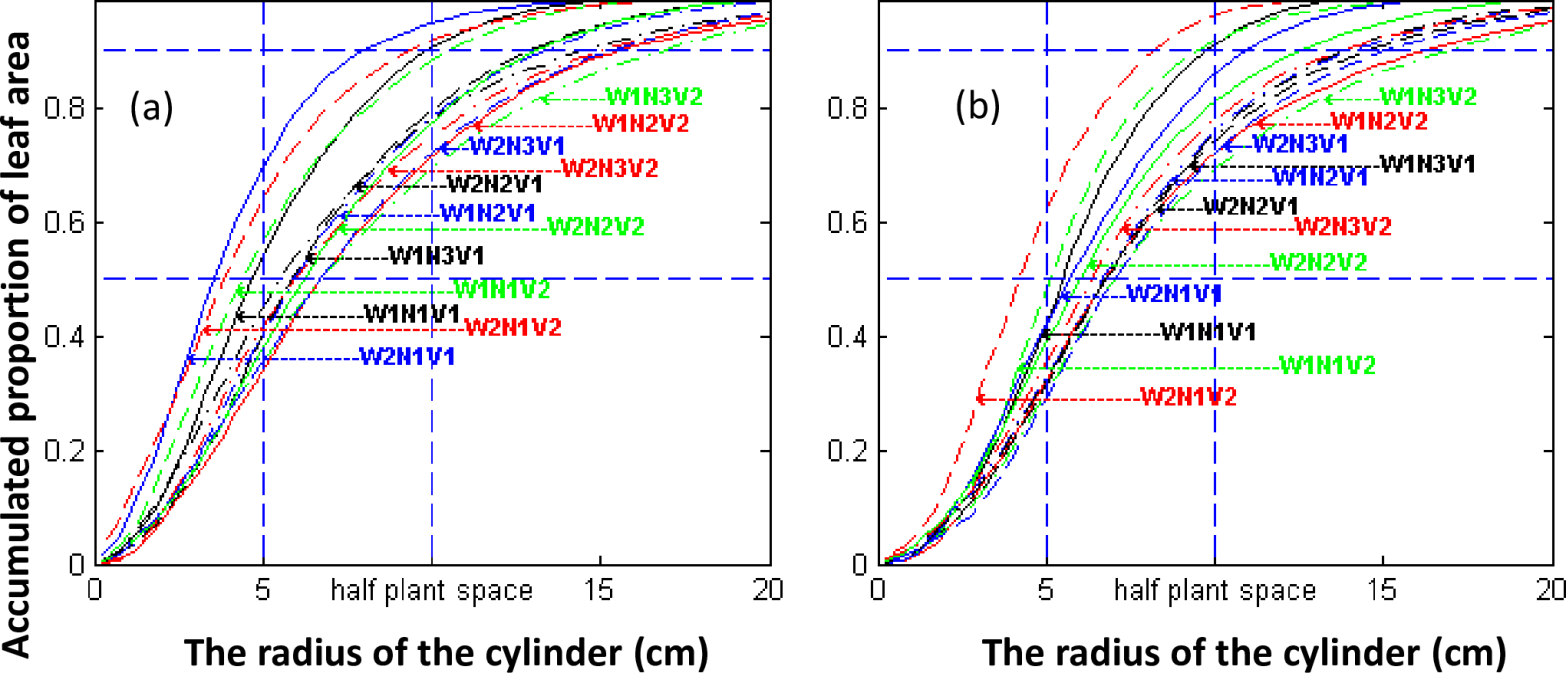
Accumulated proportion of the leaf area with the radius of the cylinder along the perpendicular-hill axis. The WiNjVk denoted the treatment which water management practice is Wi, nitrogen fertilizer application rate is Nj, variety is Vk, while the treatment number denoted by i and k varying from 1 to 3, j varying between 1 and 2. HALP (r) was derived from overlapped digital plant architecture of three representative hills. (a: Early PI; b: Last PI).

Three hills from each plot were sampled on 27-February-2015 and 10-March-2015 (the early PI and last PI stage). The system developed by Li et al [28] was used to collect data on plant architecture, construct digital plant architecture (S1 and S2 Data, S1 File), reconstruct 3D visual plant architecture (S1 and S2 Figs), accumulated leaf area index function along the Z-axis (VALAI(z)), and accumulated proportion function of the leaf area along the perpendicular-hill-axis (HALP (r)) were extracted using the virtual blade method with horizontal and cylindrical surfaces based on digital architecture [28], The VALAI(z) is leaf area on per unit ground area under the plant height z along Z-axis (Fig 1). The HALP (r) is the proportion of the leaf area in a cylinder with a radius r to the total leaf area of the hill, where hill axis was the reference for measuring the radius (Fig 2). The detail about the method described in S4 File.

### Quantitative description of plant architecture

To describe the architecture characteristics, four new architectural indices were defined based on accumulated the leaf area index along the Z-axis (β-distribution range index and the maximum density index) and accumulated proportion of the leaf area with the radius of the cylinder along the perpendicular-hill axis (α-loose index and r-compact index).

#### The maximum density index

**Fig 3:**
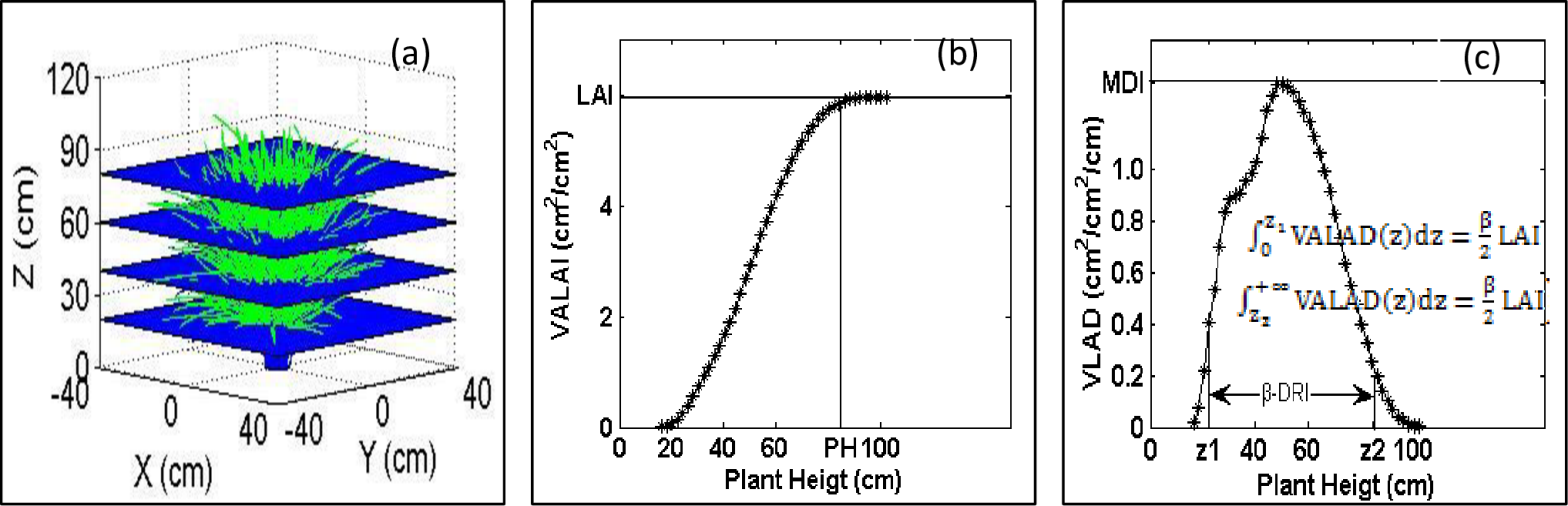
**Illustration of (a) schematic diagram of the virtual blade method with horizontal surfaces, (b) definition of the leaf area index (LAI) and plant height (PH), and (c) definition of MDI and β-DRI. PH was the plant height.** LAI is the leaf area index. MDI is the maximum density index used to identify the maximum dense leaf layer along the Z-axis. The β-DRI is β-distribution range index, i.e. the length of the two-tails interval that contains leaf area with a proportion 1-β.

The maximum density index (MDI) was used to identify the maximum density of leaf layer along the Z-axis. It is the maximum value of leaf area density on the Z-axis (VLAD (z) (Eq 1), Fig 3C). The (z) was calculated from VALAI(z) by Eq 2.

$$\text{MDI}=\max_{\text{z}}\;\text{VLAD}\;\left(\text{z}\right)$$ (1)

$$\text{VLAD}\left(\text{z}\right)=\frac{\text{dVALAI}\left(\text{z}\right)}{\text{dz}}$$ (2)

#### β-distribution range index

The β-distribution range index (β-DRI), also related to the leaf area index along the Z-axis, is defined as the length of the two-tails interval that contains a proportion 1-β of leaf area (Fig 3C), where β is the critical threshold that takes low values. The β-DRI is calculated by Eq 3 or Eq 4.

$$\beta \_\text{DRI}=\left\{{\text{z}}_{2}-{\text{z}}_{1}|\int_{0}^{{\text{z}}_{1}}\text{VALAD}\left(\text{z}\right)\text{dz}=\frac{\beta }{2}\text{LAI}\;\text{and}\;\int_{{\text{z}}_{2}}^{+\infty }\text{VALAD}\left(\text{z}\right)\text{dz}=\frac{\beta }{2}\text{LAI}\right\}$$ (3)

$$\beta \_\text{DRI}=\left\{{\text{z}}_{2}-{\text{z}}_{1}|\text{VALAI}\left({\text{z}}_{1}\right)=\frac{\beta }{2}\text{LAI and VALAI}\left({\text{z}}_{2}\right)=\left(1-\frac{\beta }{2}\right)\text{LAI}\right\}$$ (4)

For a hill, the value of β-DRI was larger if the leaf area was distributed more extensively along the Z-axis.

#### α-loose index

**Fig 4:**
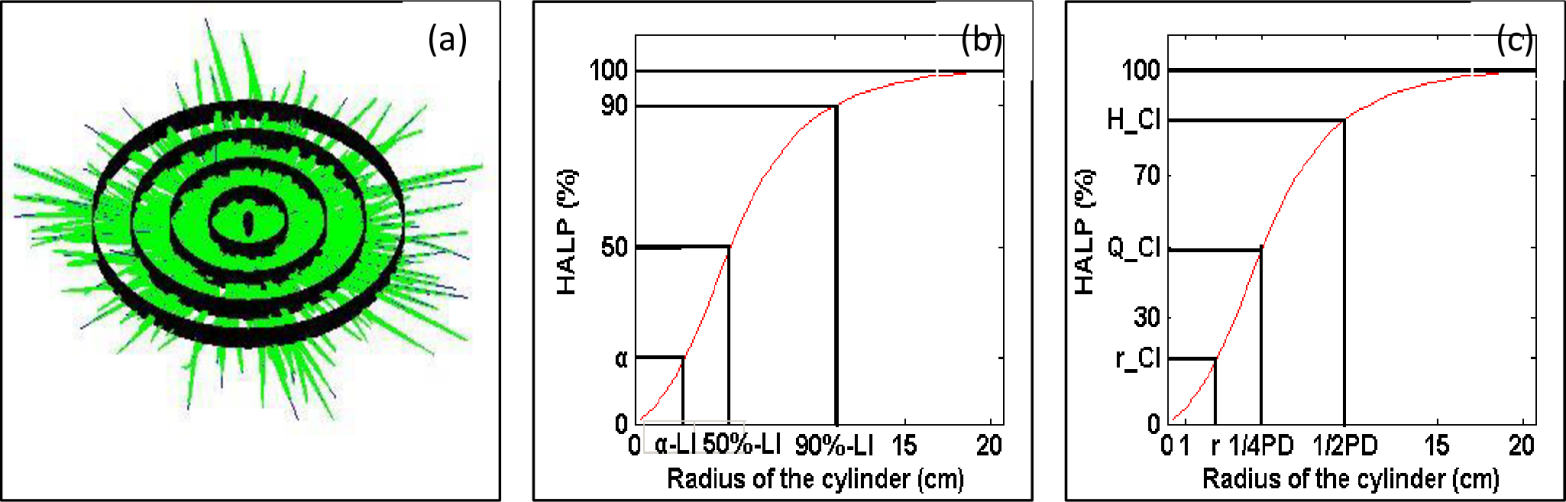
**Illustration of (a) schematic diagram of the virtual blade method with cylindrical surfaces, (b) definition of the α-loose index (α-LI), and (c) definition of the r-compact index(r-CI).** The α-loose index (α-LI) is the radius of the cylinder where the proportion of the total leaf area reaches the critical proportion α (when α is 50%, α-LI denoted by 50%-LI, while α = 90%, α-LI denoted by 90%-LI). The PD is the planting distance. The r-compact index (r-CI) is the proportion of the leaf area included in the cylinder with a radius of r (when r equals half of the planting distance, r-CI is denoted by H-CI, while r is a quarter of the planting distance, r-CI is denoted by Q-CI).

The α-loose index (α-LI) describes the degree of looseness in the plant architecture. It is defined as the radius of the cylinder where the proportion of the total leaf area reaches the critial proportion α (Fig 4C, Eq 5).

α_LI={r|HALP(r)=α} (5)

For a hill in a given α, the value of α-LI was larger if the plant architecture was looser, especially100%-LI is canopy breadth. For this particular study, 90%-LI was used to reflect the breadth of the whole plant architecture and 50%-LI was used to reflect the distribution range of half of the leaf area near the hill axis.

#### r-compact index

The r-compact index (r-CI) was used to describe the degree of compactness in the plant architecture, which was defined as the proportion of the leaf area included in the cylinder with a radius of r (Fig 4C). The r-CI was calculated by Eq 6 from HALP(r).

r_CI={α|HALP(r)=α} (6)

For a given radius r, the value of r-CI was larger if the plant architecture is more compact. For values of *r* close to the value of planting distance, r-CI is named outer compact index and reveals that neighbor hills have a lower probability of overlapping each other. When r equaled to the half of the planting distance (r-CI was denoted by H-CI), H-CI implied the overlapping proportion of neighboring hills. When r was a quarter of the planting distance (r-CI was denoted by Q-CI), Q–CI implied the degree of compactness for the plant architecture near the hill-axis.

### Statistical analysis and figure preparation

Statistical analyses were performed using analysis of variance (AOV) and paired t-Test (SPSS Statistics 17, SPSS Inc. USA). The Matlab 2009 (MathWorks USA) was used to generate figures.

## Results

### Difference analysis on plant architecture among treatments

The accumulated leaf area index along the Z-axis and the accumulated probability of the leaf area index along the perpendicular-hill-axis (VALAI(z) and HALP(r)) were derived from the digitalized plant architecture of a single hill using the virtual blade method with horizontal and cylindrical surfaces, which were also derived from the overlapped digital plant architecture of three representative hills (ODPA) (Figs 3 and 4). The architectural indices, represented by leaf area index (LAI), plant height (PH), 90%-DRI and MDI, H-CI, Q-CI, 90% -LI, and 50%-LI, were calculated from VALAI(z) and HALP(r) of ODPA and SDPA (S3 File), and these values were used for analysis of the effects of treatments by AOV and the paired t-Test (Tables 1 and 2). The analysis results indicated that

**Table 1:** The effects of experimental treatments on architectural indices (along Z-axis) on treatments, analyzed by analysis of variance (AOV) and paired t-Test. The α-loose index (α-LI) is the radius of the cylinder where the proportion of the total leaf area reaches the critical proportion α (when α is 50%, α-LI denoted by 50%-LI, while α = 90%, α-LI denoted by 90%-LI). The PD is the planting distance. The r-compact index (r-CI) is the proportion of the leaf area included in the cylinder with a radius of r (when r equals half of the planting distance, r-CI is denoted by H-CI, while r is a quarter of the planting distance, r-CI is denoted by Q-CI). Wi is water management practices, Nj is nitrogen fertilizer application rate, Vk is variety, while the treatment number denoted by i and k varying from 1 to 3, j varying between 1 and 2.

	24-Feb				10-Mar			
	LAI (%)	PH (%)	90%-DRI (%)	MDI (%)	LAI (%)	PH (%)	90%-DRI (%)	MDI (%)
**V1 vs. V2**	24.6*	6.4*	11.6*	12.0	29.4*	9.1**	9.9*	9.9
**W1 vs. W2**	56.2**	10.7**	14.0*	87.9*	50.9*	12.0**	15.2*	49.4*
**N2 vs. N1**	107.3**	27.6**	37.7**	113.9**	176.1**	28.5**	44.8**	140.7**
**N3 vs. N1**	102.1**	29.7**	39.0**	85.9**	203.7**	28.7**	40.0**	189.2**
**N3 vs. N2**	-2.1	1.8	1.0	-13.3	9.8	-0.1	-3.8	25.2
**Var×Wat**	NS	NS	NS	NS	NS	NS	NS	NS
**Var×Nit**	NS	NS	NS	NS	NS	NS	NS	NS
**Wat×Nit**	NS	NS	NS	NS	*	**	**	**
**Var×Wat×Nit**	NS	NS	NS	NS	NS	NS	NS	NS

* Significance at a 0.05 probability level.

** Significance at 0.01 probability level.

NS denotes non-significance.

**Table 2:** The effects of experimental treatments on architecture indices analyzed by paired T-test and analysis of variance. The α-loose index (α-LI) is the radius of the cylinder where the proportionof the total leaf area reaches the critial proportionα (when α is 50%, α-LI denoted by 50%-LI, while α = 90%, α-LI denoted by 90%-LI). The PD is the planting distance. The r-compact index (r-CI) is the proportion of the leaf area included in the cylinder with a radius of r (when r equals half of the planting distance, r-CI is denoted by H-CI, while r is a quarter of the planting distance, r-CI is denoted by Q-CI). Wi is water management practices, Nj is nitrogen fertilizer application rate, Vk is variety, while the treatment number denoted by i and k varying from 1 to 3, j varying between 1 and 2.

	24-Feb				10-Mar			
	H-CI (%)	5cm_CI (%)	90%- LI (%)	50% -LI (%)	H-CI (%)	Q-CI (%)	90%-LI (%)	50%-LI (%)
**V1 vs. V2**	4.1	5.0	-9.9	-5.2	-2.8	-16.1*	2.5	8.3*
**W1 vs. W2**	0.0	-3.5	8.2	10.6	0.1	-1.5	10.3*	6.3
**N2 vs. N1**	4.0**	24.2**	2.7**	-1.2**	0.9**	14.3**	13.4**	2.1**
**N3 vs. N1**	-7.3**	-10.0**	20.7**	15.2**	-6.6**	-11.1**	16.5**	10.1**
**N3 vs. N2**	-8.2	-16.4	35.8	32.5	-4.2	-10.5	19.2	14.8
**Var×Wat**	NS	NS	NS	NS	**	*	**	*
**Var×Nit**	NS	NS	NS	NS	NS	NS	NS	NS
**Wat×Nit**	NS	NS	NS	NS	NS	NS	NS	NS
**Var×Wat×Nit**	NS	NS	NS	NS	NS	NS	NS	NS

* Significance at the 0.05 probability level.

** Significance at the 0.01 probability level.

NS denotes non-significance.

(1) The LAI, PH and 90% DRI were significantly different between two varieties but LMD was not significant. Average LAI, PH and 90% DRI of NSICRc124H of all water and nitrogen treatments were higher than NSICRc222 by 24.6%, 6.4% and 11.6% at the early PI and 29.4%, 9.1% and 9.9% at the last PI, respectively. However, LAI of NSICRc124H (V1) was lower than fully irrigated NSICRc222 (V2) in the lowland and zero N (W1N1) at last PI, 90% DRI of fully irrigated NSICRc124H (V1) was lower than NSICRc222 (V2) in the lowland and zero N (W1N1) at last PI and with the alternative water and dry conditions in the upland and middle N (W2N2) at early PI.

(2) The difference in LAI, PH, 90%-LI and MDI was significant between the two water management schemes. The average LAI, PH, 90%-DRI and MDI of all varieties and nitrogen management were higher in fully irrigated in the lowland (W1) than those in alternative water and dry conditions in the upland (W2) by 56.2%, 10.7%, 14.0% and 87.9% at early PI and 50.9%, 12.0%, 15.2% and 49.4% at last PI, respectively. However, LAI, PH, 90%-DRI and MDI in the fully irrigated the lowland (W1) were lower than the alternative water and dry conditions of the upland (W2) under two of the lower nitrogen management conditions (zero N and NSICRc124H (N1V1), zero N and NSICRc222 (N1V2) at the early and last PI stages.

(3) The difference in LAI, PH, 90%-DRI and MDI was significant between zero N (N1) and middle N (N2), also zero N (N1) and high N (N3), but not between middle N (N2) and high N (N3). The average LAI, PH, 90%-DRI and MDI of all varieties and water management strategies were higher in middle N (N2) than zero N (N1) by 107.3%, 27.6%, 37.7% and 113.9% at early PI and 176.1%,28.5%,44.8% and140.7% at last PI, respectively, and higher in high N (N3) than zero N (N1) by 102.1%, 29.7%, 39.0% and 85.9% at early PI, and 203.7%, 28.7%, 40.0% and 189.2%at last PI, respectively.

(4) The interacting effects of cultivar and water and nitrogen management on LAI, PH, 90%-DRI and MDI almost were not significant. However, the effects of Water×Nitrogen on LAI, PH, 90%-DRI and MDI were significant. This outcome suggested that limited one of water and nitrogen was the determinator of plant growth.

(5) The H-CI, Q-CI, 90% -LI, 50%-LI were not significantly different at a p = 0.05 level between two varieties, except Q-CI and 50%-LI at last PI.

(6) The H-CI, Q-CI, 90% -LI, 50%-LI were not significantly different between two water management practices, except 90% -LI at last PI.

(7) The H-CI, Q-CI, 90%-LI, 50%-LI were significantly different among the three nitrogen management conditions, according to AOV, between zero N (N1) and middle N (N2), also zero N (N1) and high N(N3), but not significant between middle N (N2) and high N (N3). Average H-CI, Q-CI, 90%-LI and 50%-LI of all varieties and water management were higher in the middle N (N2) than zero N (N1) by 4.0%, 24.2%, 2.7% and -1.2% at early PI and 0.9%, 14.3%,13.4% and 2.1% at last PI, and higher in high N (N3) than zero N (N1) by -7.3%, -10.0%, 20.7% and 15.2% at early PI and -6.6%, -11.1%, 16.5% and 10.1%at last PI.

(8) The effects of interaction factors on H-CI, Q-CI, 90%-LI and 50%-LI almost were not significant. However, the effects of Var×Wat on H-CI, Q-CI, 90%-LI and 50%-LI were significant. This result suggested the effects of variety were limited by water stress.

### Trend analysis of the effects of treatments on plant architecture

**Fig 5:**
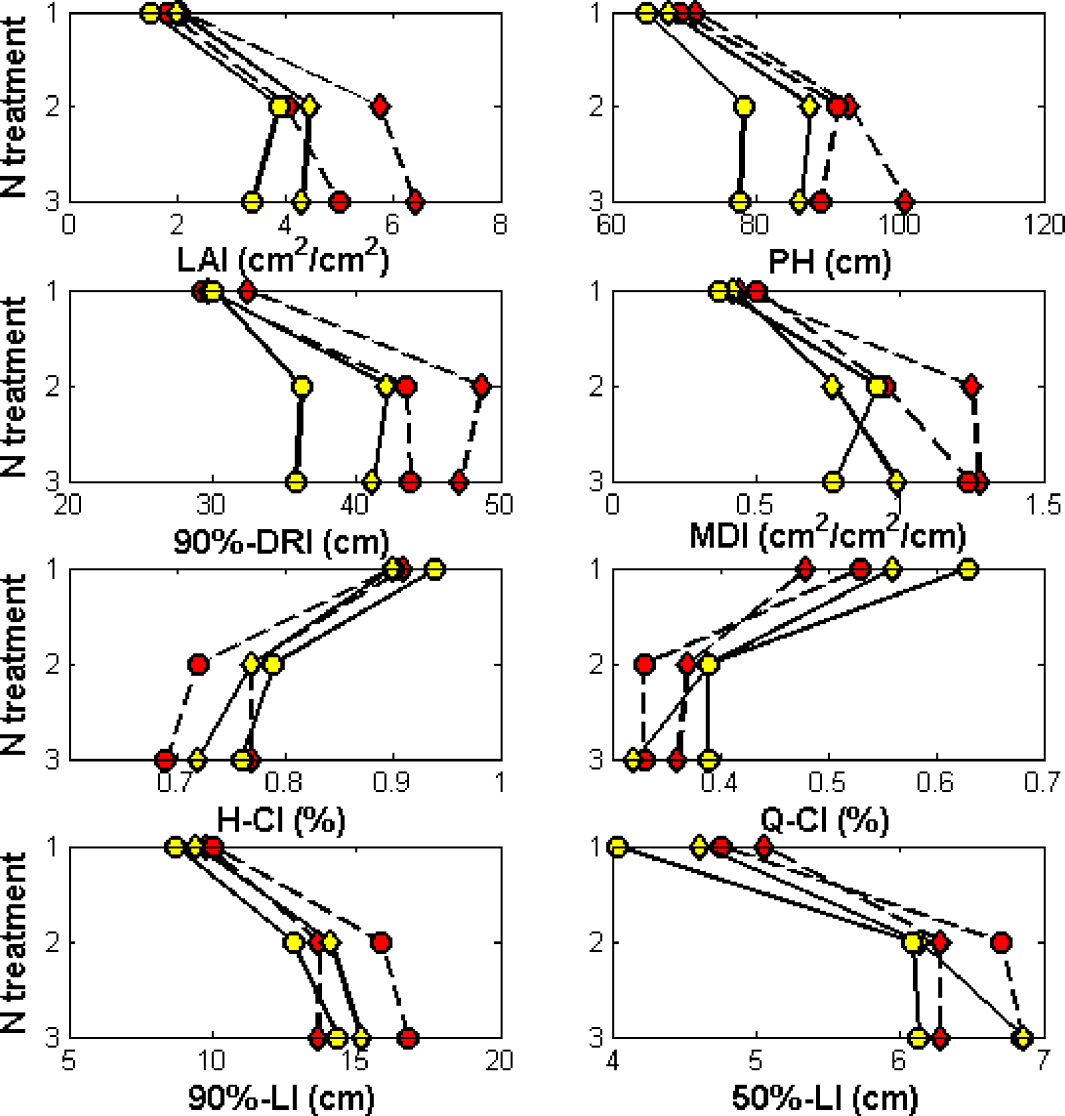
Trend of the effects of treatments on plant architecture. N:1-N1,2-N2,3-N3, WiNjVj denoted the treatment with Wi for water management practices varying from W1 to W3, Nj for nitrogen fertilizer application rate varying from N1 to N3, and Vk for variety as V1 and V2. HALP(r) was derived from overlapped digital plant architecture of three Neighbor hills.

The architectural indices, i.e., LAI, PH, 90%-DRI and MDI, H-CI, Q-CI, 90% -LI, 50%-LI in the period from early to last PI were calculated at two stages. The analysis results are illustrated in Fig 5.

(1) As the amount of nitrogen fertilizer increased, LAI and PH increased for the fully irrigated samples in the lowland (W1), and increased then decreased for the alternative water and dry samples in the upland (W2), 90%-DRI increased then decreased, and DMI of NSICRc222 increased for the fully irrigated in the lowland samples (W1) and increased then decreased for the alternative water and dry samples in the upland (W2). Those results contrasted the results for NSICRc124H. H-CI and Q-CI decreased, which contrasted the results for 90%-LI and 50%-LI.

(2) Compared to NSICRc222, NSICRc124H had larger LAI, PH and 90%-DRI, larger H-CI under fully irrigated conditions in the lowland (W1) and smaller H-CI under alternative water and dry conditions in upland (W2). NSICRc124H also had a larger 90%-LI for fully irrigated samples in the lowland (W2), a smaller 90%-LI for alternative water and dry conditions in the upland (W1), larger 50%-LI under fully irrigated conditions in the lowland (W2), and smaller 50%-LI under alternative water and dry conditions in the upland (W1), except with zero N and samples that were fully irrigated in the lowland (N1W1). There is no significant trend in DMI and Q-CI according to water management strategy.

(3) Compared to samples under alternative water and dry conditions in the upland (W2), LAI, PH, 90% -DRI and DMI were larger under fully irrigated conditions in the lowland (W1), 50%-LI was larger, and Q-CI was smaller, except for NSICRc124H with high N (N3). There was no significant trend of H-CI and 90%-LI affected by different water management strategies.

### Correlation relationship among AIs

The relationships among the indices were investigated by correlation analysis. The Pearson coefficients and significance among the averaged indices of both stages are shown in Table 3. The results indicated that the Pearson correlations among the eight indices were significant. The Pearson correlations among LAI, PH, 90%-DRI and MDI and among H-CI, Q-CI, 90% -LI, 50%-LI were over 0.9. Therefore, the eight architectural indices could be classified into two groups: LAI, PH, 90%-DRI and MDI as vertical architecture indices and H-CI, Q-CI, 90% -LI, 50%-LI compact architecture indices.

**Table 3:** Pearson coefficients among the indices and accumulated biomass. The α-loose index (α-LI) is the radius of the cylinder where the proportion of the total leaf area reaches the critical proportion α (when α is 50%, α-LI denoted by 50%-LI, while α = 90%, α-LI denoted by 90%-LI). The PD is the planting distance. The r-compact index (r-CI) is the proportion of the leaf area included in the cylinder with a radius of r (when r equals half of the planting distance, r-CI is denoted by H-CI, while r is a quarter of the planting distance, r-CI is denoted by Q-CI).

	LAI	PH	90%-DRI	MDI	H-CI	Q-CI	90%-LI	50%-LI
**LAI**	1	.966**	.962**	.961**	-.803**	-.831**	.771**	.826**
**PH**	.966**	1	.974**	.923**	-.821**	-.859**	.806**	.849**
**90%-DRI**	.962**	.974**	1	.930**	-.816**	-.837**	.803**	.837**
**MDI**	.961**	.923**	.930**	1	-.856**	-.861**	.828**	.866**
**H_CI**	-.803**	-.821**	-.816**	-.856**	1	.954**	-.994**	-.980**
**Q_CI**	-.831**	-.859**	-.837**	-.861**	.954**	1	-.942**	-.993**
**90% LI**	.771**	.806**	.803**	.828**	-.994**	-.942**	1	.968**
**50% LI**	.826**	.849**	.837**	.866**	-.980**	-.993**	.968**	1

* Significance at the 0.05 probability level.

** Significance at the 0.01 probability level.

NS denotes non-significance.

### The effects of AIs on accumulated biomass

**Fig 6:**
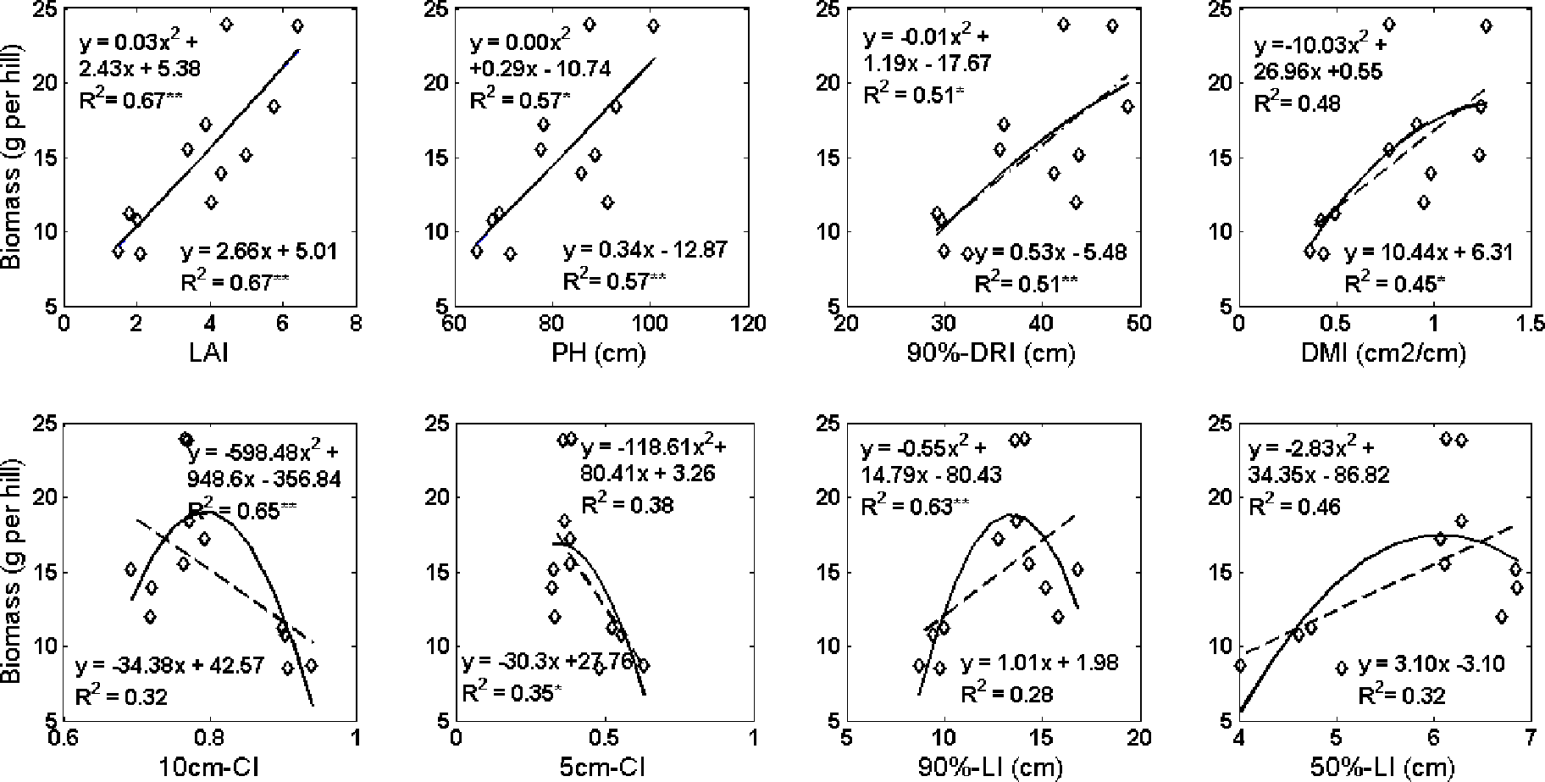
Regression analysis of the relationship between accumulated biomass and the eight indexes.

The relationship between accumulated biomass and the eight indexes were investigated by regression analysis (Fig 6). The results showed that (1) there were significant linear relationships between accumulated biomass and LAI, PH, 90%-DRI, or DMI, and a quadratic relationship between accumulated biomass and H_CI,Q-CI, 50%-LI or 90% LI, but the relationships were not significant for Q-CI and 50%-LI, and (2) The 0.79 of H-CI and 13.45cm of 90%-LI were the optimum values for biomass accumulation if the plant distance was 10cm.

### Better plant architecture for higher biomass accumulation

The four plant architectures with biomass in the top third were selected as the better architectures. Analysis from Table 4 combined with the results of section 3.4 suggested that:

**Table 4:** Data of accumulated biomass and architecture indices (AIs) for the better architectures selection. The Biom is accumulated biomass, Rel Bios is the type of relationship between accumulated biomass, LPC is a positive linear correlation, QC is a quadratic correlation, Lower B is the lower bound of AIs of good plant architecture, and Upper B is the lower bound of AIs of good plant architecture. The α-loose index (α-LI) is the radius of the cylinder where the proportion of the total leaf area reaches the critical proportion when is 50%, α-LI denoted by 50%-LI, while 90%, α-LI denoted by 90%-LI. The PD is the planting distance. The r-compact index (r-CI) is the proportion of the leaf area included in the cylinder with a radius of r (when r equals half of the planting distance, r-CI is denoted by H-CI, while r is a quarter of the planting distance, r-CI is denoted by Q-CI). WiNjVj denotes the treatment with Wi for water management practices varying from W1 to W3, Nj for nitrogen fertilizer application rate varying from N1 to N3, and Vk for variety as V1 and V2. HALP(r) was derived from overlapped digital plant architecture of three Neighbor hills.

	Biom	LAI	PH	90% D	DMI	H-CI	Q-CI	90%-LI	50%-LI
**W1N1V1**	8.5	2.11	71.62	32.49	0.44	0.91	0.48	9.79	5.06
**W1N1V2**	11.26	1.82	69.37	29.26	0.5	0.9	0.53	9.99	4.75
**W1N2V1**	18.39	5.77	93.07	48.75	1.25	0.77	0.37	13.7	6.29
**W1N2V2**	11.96	4.04	91.31	43.46	0.95	0.72	0.33	15.86	6.71
**W1N3V1**	23.87	6.43	100.9	47.23	1.28	0.77	0.36	13.67	6.29
**W1N3V2**	15.12	5	88.86	43.75	1.24	0.69	0.33	16.81	6.85
**W2N1V1**	10.73	2.03	67.92	29.73	0.42	0.9	0.56	9.43	4.62
**W2N1V2**	8.7	1.5	64.65	30.06	0.37	0.94	0.63	8.73	4.03
**W2N2V1**	23.95	4.47	87.65	42.19	0.77	0.77	0.39	14.12	6.14
**W2N2V2**	17.18	3.9	78.3	36.18	0.92	0.79	0.39	12.81	6.09
**W2N3V1**	13.95	4.32	86.13	41.2	0.99	0.72	0.32	15.19	6.86
**W2N3V2**	15.47	3.39	77.72	35.76	0.77	0.76	0.39	14.37	6.13
**Rel Bios**		LPC	LPC	LPC	LPC	QC	QC	QC	QC
**Lower B**	23.95	6.43	100.9	48.75	1.28	0.79	0.39	14.12	6.29
**Upper B**	17.18	3.90	78.3	36.18	0.77	0.77	0.36	12.81	6.09

(1) The relationship of LAI, PH, D 90%, DMI to dry matter accumulation is a positive linear relationship, and larger LAI, PH, D, and 90% DMI corresponded to a large accumulation of dry matter. Plant architecture with high dry matter accumulation had LAI≥3.9, PH≥ 78.3, 90% DRI≥ 36.18 and DMI ≥0.77.

(2) The relationship of H-CI, Q-CI, 90% -LI, 50% -LI with dry biomass accumulation was quadratic, and moderate H-CI, Q-CI, 90% -LI, 50% -LI corresponded to greater dry matter accumulation. Plant architecture with high dry matter accumulation had 0.79≥H-CI≥0.77, 0.39≥Q-CI≥0.36, 14.12≥90% -LI≥12.81 and 6.29≥50% -LI≥6.09.

(3) A Larger leaf area index did not always correspond to higher dry matter accumulation, and a combination of larger leaf area index and the suitable compact degree was sufficient and necessary for higher dry biomass accumulation. Plants with LAI≥3.9 and 0.79≥H-CI≥0.77(or 0.39≥Q-CI≥0.36, 14.12≥90%- LI≥12.81, or 6.29≥50%- LI≥6.09) had higher dry matter accumulation.

(4) In this study, W2N2V2, W1N2V1, W1N3V1, W2N2V1 could build relatively good plant architecture with a relatively high dry biomass accumulation.

## Conclusion and discussion

In this paper, eight indices were derived from 3D digital plant architecture to quantitatively describe plant architecture. Then, the indices were applied to analyze the effects of the varieties and water and nitrogen management on the relationship to biomass accumulation. These indices followed conclusions that were derived from this study.

1. The architecture indices were closely related to accumulated biomass. Biomass accumulation has a positive linear correlation with vertical architecture indices, and has a quadratic correlation with compact architecture indices, respectively (Fig 6). The R^2^ of H-CI and 90%-LI (0.65, 0.63) were close to LAI (0.67) which widely used in agronomic and breeding practices [6,24], which suggested the H-CI and 90%-LI would become the most important index to quantify plant architectural compactness.
2. The combination of larger vertical AI with appropriate compact AI was sufficient and necessary for better plant architecture and to have higher dry biomass (Table 4). A moderate degree of compactness was an advantage for accumulating higher biomass, which agreed with the rice breeding approach of choosing a moderate compact plant-type [27].
3. Varieties, water, nitrogen management and the interaction of water and nitrogen significantly affected vertical architecture indices, and compact architecture indices were influenced by the nitrogen management interaction factors of Var×Wat. Furthermore, there were trends in the influence of varieties, water, and nitrogen management on AIs (Fig 5). Thus, varieties, water and nitrogen management could be used to adjust plant architecture for higher accumulated biomass.

These architecture indices provide a new way to quantitatively describe and model the relationship between plant structure and function. However, the effects of agronomy management on these indices and the relationship of these indices to biomass accumulation are complex [20]. The results of this study need further testing, and these indices need further exploration, for example, how do the AIs affect the biomass accumulation during the period from HD to PM and at the sampling stage, and rice yield, which is the best index to be used in the breeding practices for ideal plant type, and in agronomic practices for optimizing water manager, Nitrogen fertilizer application, and planting space.

## Supporting information

S1 Data Digital plant architecture. This file is Matlab data file, which corresponds to the digital plant architecture sampled on 24 Feb. Each cell includes 10 variables: Radius, TurnplateH, EarthDepth, H1 and H2 are the parameters of measuring apparatus, StemNum is the tiller number of the hill, SSP_LCAA correspond to the special position of the stem, LeafCurves correspond to leaf midrib curves, LeafShapes correspond to leaf shape.
(MAT)

S2 Data Digital plant architecture. This file is Matlab data file, include two cell arrays, corresponds to the digital plant architecture sampled on 24 Feb and 10-Mar. Each cell includes 10 variables: Radius, TurnplateH, EarthDepth, H1 and H2 are the parameters of measuring apparatus, StemNum is the tiller number of the hill, SSP_LCAA correspond to the special position of stem, LeafCurves correspond to leaf midrib curves, LeafShapes correspond to leaf shape.
(MAT)

S1 File Code for computing the accumulated leaf area index along the Z-axis and Accumulated proportion of the leaf area index along the perpendicular-hill axis. The mainVertical.m and mainCylinder.m are runnable file.
(RAR)

S2 File Biomass data. (XLSX)

S3 File Plant architecture indices. The architectural indices, i.e., LAI, PH, 90%- DRI and MDI,H-CI, Q-CI, 90% -LI, and 50%-LI were calculated from VALAI(z) and HAPLAIHALP(r) of SDPA.
(XLSX)

S4 File Digital construction and trait extraction of plant architecture. The steps to collect data on plant architecture, construct digital plant architecture, reconstruct 3D visual plant architecture and the leaf area distribution of plant architecture using the system developed by Li.
(DOCX)

S1 Table Abbreviations. Abbreviations of indices and phrases are listed with their full words and meaning.
(DOCX)

S1 Fig Visual plant architecture. The Visual plant architecture sample on 24 Feb.
(TIF)

S2 Fig Visual plant architecture. The Visual plant architecture sample on 10-Mar.
(TIF)
