# Supplementary material for: Quantitative descriptions of rice plant architecture and their application
Source: PLoS One. 2017 May 17;12(5):e0177669. doi: 10.1371/journal.pone.0177669 (PMC5435225; doi:10.1371/journal.pone.0177669)
Supplement: S4 File — The steps to collect data on plant architecture, construct digital plant architecture, reconstruct 3D visual plant architecture and the leaf area distribution of plant architecture using the system developed by Li. (DOCX) [file pone.0177669.s006.docx]

Digital construction and trait extraction of plant architecture

Three hills from each plot were sampled on 27-February-2015 and 10-March-2015 (the early PI and last PI stage).The system developed by Li was used to collect data on plant architecture, construct digital plant architecture, reconstruct 3D visual plant architecture and the leaf area distribution of plant architecture using the following steps.

(1)The data on plant architecture was collected based on the location-separation–measurement method. Spatial position and azimuth of a leaf were measured with a Cylindrical Coordinate graph. The data on leaf shape and midrib curve were derived from images of tillersand leaves captured by image acquisition equipment.

(2) The digital plant architecture, as structural data with a spatial position reference, shape and size for different objects from hills in the field to stems and leaves attached to the plant, was constructed as follows:

| *For Field: planting space; sampled plant number (****pm****); variety; nitrogen level; water level*  *For hill: hill position in the field*  *For tiller: stem spatial position, length and radius*  *For leaf: leaf node, azimuth, shape and venation curve* |
| --- |

(3) The 3D visualization of a plant in the field was reconstructed in the visualization of geometrical structure on digital architecture with the stem represented as a cylinder and leaves represented as a wireframe surface.

(4) Accumulated leaf area index along the z-axis and accumulated proportion of the leaf area along the perpendicular-hill-axis (VALAI(z) and HALP (r))were extracted with the virtual blade method using horizontal and cylindrical surfaces based on digital architecture (Figs 1-2), in which z was the height above land surface and r was the radius of the cylinder with hill axis as the center axis.
