# Supplementary material for: Quantitative descriptions of rice plant architecture and their application
Source: PLoS One. 2017 May 17;12(5):e0177669. doi: 10.1371/journal.pone.0177669 (PMC5435225; doi:10.1371/journal.pone.0177669)
Supplement: S1 Table — Abbreviations of indices and phrases are listed with their full words and meaning. (DOCX) [file pone.0177669.s007.docx]

| **Abbreviation** | **Full Words** | **Meaning** |
| --- | --- | --- |
| AI | Architectural index | Architectural index |
| LAI | Leaf area index | Leaf area index |
| PH | Plant height | Plant height |
| β-DRI | β-distribution range index |  |
| α-LI | α-loose index | It characterizes the loose degree of plant architecture and is defined as the radius of the cylinder that contains a proportion α of the total leaf area. |
| 90%-LI | 90%-loose index | As α-LI, where α=90%. |
| 50%-LI | 50%-loose index | As α-LI, where α=50%. |
| r-CI | r-compact index | It describes the compact degree of plant architecture and is defined as the proportion of leaf area of a hill included in a cylinder centered at the hill center and with a radius of r |
| H-CI | Half-compact index | As r-CI, where r=half plant space. |
| Q-CI | Quarter-compact index | As r-CI, where r= quarter plant space. |
| MDI | the maximum density index | It is defined as the maximum value of leaf area density on the z-axis. |
| V1 | NSICRc124H | A hybrid rice variety. |
| V2 | NSICRc222 | A inbred rice variety. |
| W1 | fully irrigated in lowland | fully irrigated in lowland |
| W2 | alternative water and dry at upland | alternative water and dry at upland |
| N1 | nitrogen fertilizer application rate (N1: 0kg ha^−1^) | nitrogen fertilizer application rate (N1: 0kg ha^−1^) |
| N2 | nitrogen fertilizer application rate (N2:170 kg ha^−1^) | nitrogen fertilizer application rate (N2:170 kg ha^−1^) |
| N3 | nitrogen fertilizer application rate (N3:240 kg ha^−1^) | nitrogen fertilizer application rate (N3:240 kg ha^−1^) |
| PI | the panicle initiation | the panicle initiation |
| VALAI(z) | Accumulated leaf area index along the z-axis | Accumulated leaf area index along the z-axis |
| HALP (r) | Accumulated proportion of the leaf area along the perpendicular-hill- axis | Accumulated proportion of the leaf area along the perpendicular-hill- axis |
| VLAD(z) | value of leaf area density on the z-axis | value of leaf area density on the z-axis |
