# Supplementary figures and images for: Quantitative descriptions of rice plant architecture and their application
Source: PLoS One. 2017 May 17;12(5):e0177669. doi: 10.1371/journal.pone.0177669 (PMC5435225; doi:10.1371/journal.pone.0177669)

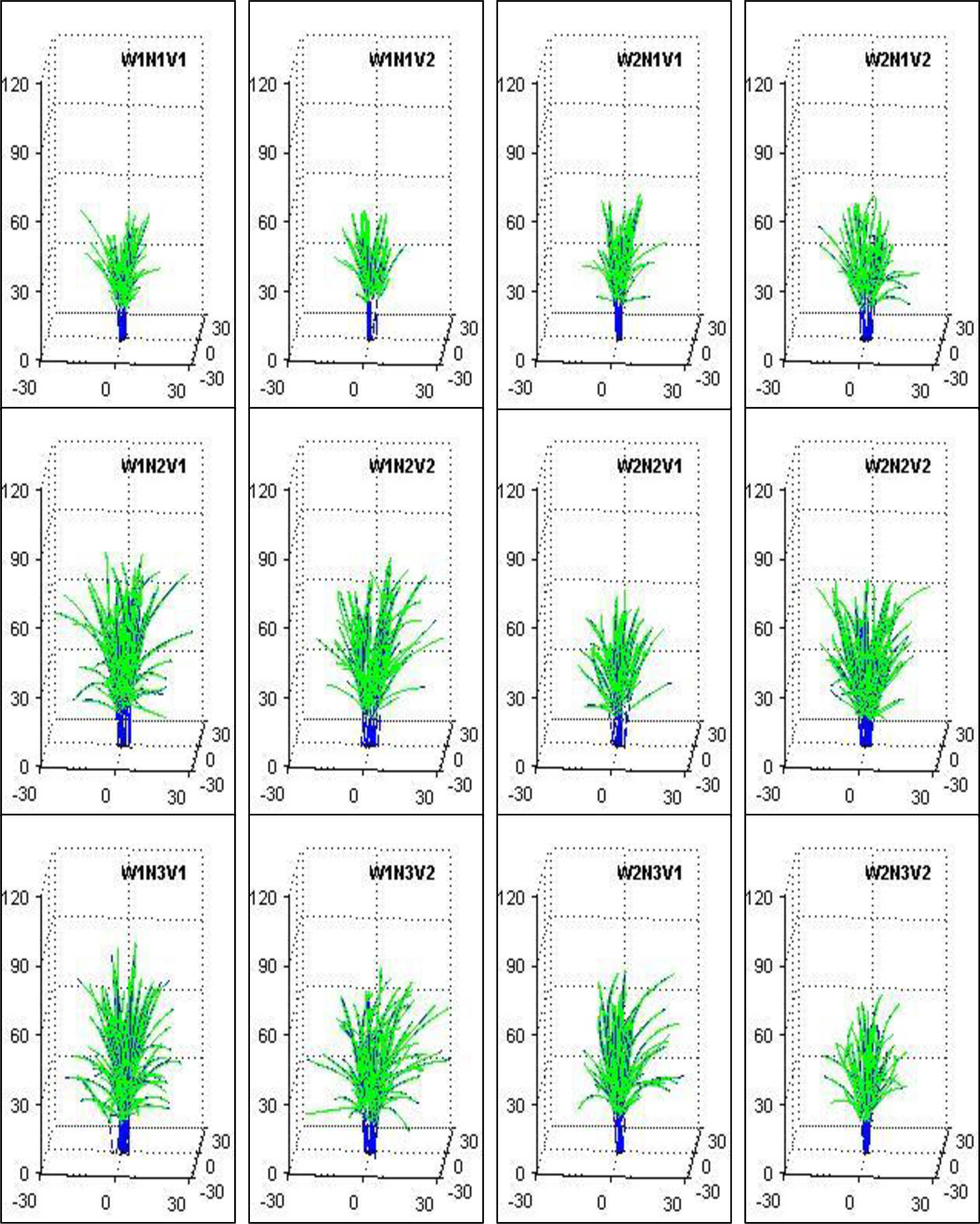

Supplement: S1 Fig — The Visual plant architecture sample on 24 Feb. (TIF) [file pone.0177669.s008.tif]

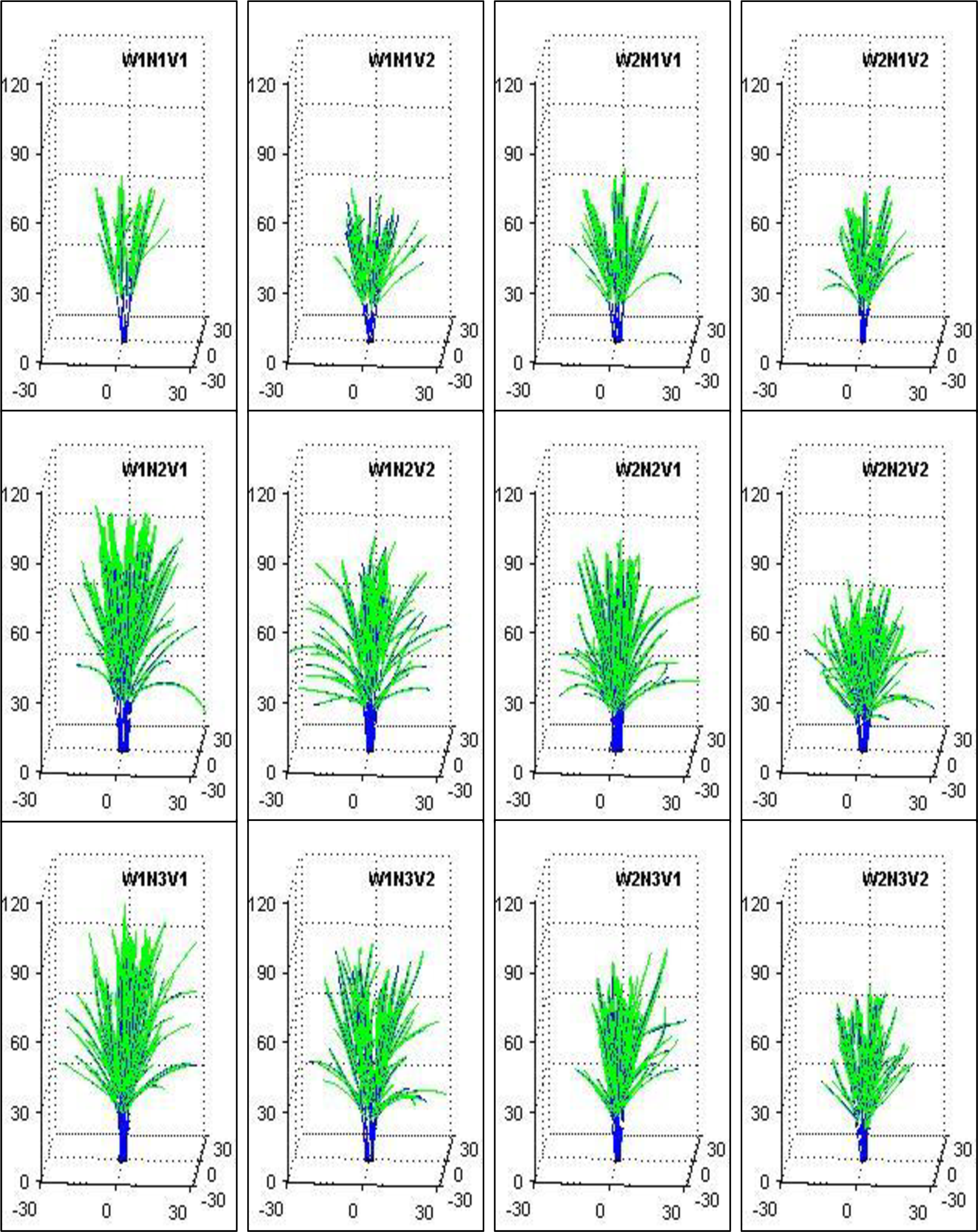

Supplement: S2 Fig — The Visual plant architecture sample on 10-Mar. (TIF) [file pone.0177669.s009.tif]
